# Shared Decision-Making and Patient-Reported Outcome Measures in Valvular Heart Disease

**DOI:** 10.3389/fcvm.2022.863040

**Published:** 2022-04-08

**Authors:** Sahrai Saeed, Elisabeth Skaar, Andrea Romarheim, John B. Chambers, Øyvind Bleie

**Affiliations:** ^1^Department of Heart Disease, Haukeland University Hospital, Bergen, Norway; ^2^Cardiothoracic Centre, Guy's and Saint Thomas' Hospital, London, United Kingdom

**Keywords:** aortic stenosis, aortic valve replacement, frailty, patient-reported outcome measures, quality of life, shared decision-making, transcatheter aortic valve implantation, valvular heart disease

## Abstract

Patient-centered health care emphasizes shared decision-making (SDM), incorporating both clinical evidence and patient preferences and values. SDM is important in heart valve disease, both because there might be more than one treatment option and due to the importance of adherence after intervention. We aimed to describe patient information and involvement in decision-making about care and recording of patient-reported outcome measures (PROMs) in valve interventions. The opinion piece and recommendations are based upon literature review and our own experience from specialist valve clinics. Before a valve intervention, adequate patient information, discussion of the various treatment options and exploring patient preferences, in line with the concept of SDM, may improve post-intervention quality of life. After intervention, patients with prosthetic heart valves require adequate counseling and close follow-up to make them more confident and competent to manage their own health, as well as to maintain the efficacy of treatment provided. PROMs inform SDM before and improve care after valve intervention, focusing on outcomes beyond mortality and morbidity. SDM may improve post-intervention quality of life. Formal PROMs questionnaires inform SDM, quantify patient centered changes and should be used more often in clinical practice and research. A thorough assessment of baseline frailty status in patients scheduled for valve intervention is essential and may affect postoperative outcome.

## Introduction

Health policy makers encourage patient-centered health care including shared decision-making (SDM) (https://www.bhvs.org.uk/bhvs-blueprint/). SDM is a collaborative process involving at least a healthcare professional and a patient, where both participate in decision-making (1). The goal is to reach a consensus of decision incorporating best available evidence and patient priorities (2). The purpose of SDM is also to keep a balance in power between patients and physicians or other caregivers (1), and to replace the more traditional authoritarian communication models, in order to reach decisions consistent with patients' goals of care. Evolving from the original focus, SDM also encompasses management, self-care and lifestyle changes (3). In valvular heart disease (VHD), for which surgery or transcatheter interventions are common, this approach can be divided into care before and after the intervention. Before the procedure, there must be adequate information of the patient, discussion of the various treatment options and actively seeking patients' preferences and involvement. After the valve intervention, sufficient information should be provided for the patient in order to support self-management and take care of their own health. This is particularly important in the period of time after receiving treatment at hospital in order to maintain the efficacy of treatment provided. Patients are also expected to take more responsibility for their own health and get actively involved in the disease management. After the procedure, the patient-centered approach goes beyond the traditional measures of mortality and morbidity to assess patient-reported outcome measures (PROMs).

The aims of this review are to describe: (1) SDM with focus on patient information and involvement in decisions about care and; (2) commonly used instruments for recording PROMs after interventions.

## Shared Decision-Making Before Intervention

In clinical care, most patients appreciate SDM (4), which alongside careful baseline risk stratification is important for better outcomes after surgical aortic valve replacement (SAVR) (2, 5). Treating depression and modifying negative illness beliefs before surgical intervention may further improve outcomes in these patients (5). Patient preference is cited as the first indication in choosing a biological instead of a mechanical valve for the younger patient (5). However, this choice is made based upon the mutual relationship between patient preferences and medical practice, especially after providing adequate information by the physician or other healthcare professional regarding the two available options: (1) mechanical which are thrombogenic and require lifelong anticoagulation; and (2) biological which has shorter durability and carries risk of degeneration and reoperation in younger patients. Indeed, the 2020 American College of Cardiology (ACC)/American Heart Association (AHA) guidelines on VHD highlight including the patient's values and preferences and the indications for and risks of anticoagulant therapy when making a decision about surgery (6). Similarly, current European guidelines for the management of VHD (7), also reinforce the critical role of the patient's involvement in the mode of intervention, beyond the Heart Teams integration of the clinical, anatomical, and procedural characteristics and conventional scores. Most patients can expect a significant improvement in survival, symptoms, exercise tolerance and disease specific quality of life (QoL) after AVR, but their physical QoL may not return to normal. However, in patients <60 years, mental QoL after biological AVR was significantly better than age-matched control subjects (8), highlighting the importance of pre-intervention SDM in prosthetic valve selection, particularly in younger patients.

Evidence suggests that transcatheter aortic valve implantation (TAVI) compared with conservative treatment improves QoL, symptoms and physical function related to aortic stenosis (AS). However, the psychological or general health benefits appear to be modest (9), particularly if valve intervention is offered late. Frailty, a known predictor of adverse outcome, is defined as a state of reduced physiological reserve and diminished resistance to stressors. There is no consensus on the definition, and the two main models are the accumulation of deficits (adding together an individual's number of impairments and condition) creating a Frailty Index (10) and the specific physical phenotype consisting of 5 possible components (weight loss, exhaustion, weakness, slowness, and reduced physical activity (11).

Older adults, especially those with multiple chronic conditions and frailty, may have different goals of care than younger healthier adults. There may be less focus on survival and more on QoL, including physical function and independence (3). High-risk elderly patients with severe AS being evaluated for TAVI, can define their goals through a simple question “What do you hope to accomplish by having your valve replaced?” (12). Repairing the aortic valve if AS is one of several comorbid conditions may not restore a patient's functional status and QoL (9, 13). SDM does not justify patients demanding futile treatment. The decision to offer valve intervention should be made by the heart valve team, weighing benefit vs. risk, while taking into account comorbidities, life-expectancy, frailty, procedural risk and symptom burden.

Patient preference is the most common reason for selecting medical management in severe symptomatic AS (14). However, patients receiving medical management received less information and felt less engaged by their heart valve physicians than those receiving TAVI or SAVR (14).

A core aim of a valve clinic is to inform patients adequately before intervention is required (15). Exploring patient preferences and values during this process enables SDM over many visits and not during a single consultation immediately before the intervention. However, providing sufficient information is challenging, and there is only patchy availability of reliable literature (15, 16). Some patients may turn first to the internet, but sites may be biased commercially or toward the specialty of the hospital preparing the website (17). A patient's understanding of valve disease may therefore be limited (18–20). In surveys, <20% were aware of heart valve disease and only 7% knew what AS was (19, 20).

Decision aids may improve a patient's understanding of available treatment options (21). These may be written material, charts, graphs presented electronically or as brochures available at hospitals or general practitioner (GP) offices. An experienced patient voluntarily affiliated with a hospital may help new patients (22). Patient preferences can be formalized using questionnaires based on PROMs (18, 23–25). Overall, in health care delivery systems, PROMs-based information is underused, probably because of the perceived lack of time in the clinic (23), and lack of effective educational tools for risk communication. PROMs facilitate symptom monitoring, and improve patient-doctor communication and could be used in individuals or groups (23). Making the PROMs score available through the electronic patient record and dashboards before consultation and preferably to show any changes over several visits, is expected to increase awareness among doctors about the individual patient's needs. Patient-reported health status seems to be a useful supplement to the established physical examination during the clinical assessment (26), and may improve risk assessment of patients with AS. Aggregated PROMs scores could be used before intervention to guide patients on their likely outcomes beyond mortality and morbidity (23). In some countries it has become mandatory to collect and report PROMs for surgical interventions. However, decision aids, written material and brochures are not a substitute for direct communication between the physician and older patients with multiple conditions (for example reduced cognition or frailty).

## Patient-Reported Outcome Measures (Proms) After Intervention

The main objectives in treating VHD is to decrease the rate of premature death and improve clinical outcomes including QoL. Both SAVR and TAVI lead to recovery of LV function, LV mass regression and improved survival and symptoms. However, QoL is not routinely assessed and this is obviously of paramount importance to the patient. PROMs represents a strategy of evaluating health status by the patients themselves, for example assessing QoL after SAVR and TAVI (Table 1) (27–29), which require the use of optimal QoL instruments. There is, therefore, increasing work on PROMs.

**Table 1:** List of commonly used Questionnaires to assess patient-reported outcome measures (PROMs) as useful aids in shared-decision making in surgical practice and other areas of therapeutic medicine.

**No**	**Type of instruments**	**Health status assessed**
1	The Minnesota Living with Heart Failure Questionnaire (MLHFQ). Includes 21 formal questions and describes physical, emotional and socioeconomic aspects of quality of life related to a specific disease. Emotional (five questions; range 0–25), physical (eight question; range 0–40).	Cardiac- or disease-specific
2	Medical Outcomes Study Short Form-12 (SF-12) questionnaire. Includes 12 items capturing eight domains of self-rated health status of physical component summary (PCS) and a mental component summary (MCS).	Generic
3	EuroQol 5 Domains (EQ-5D) Provides information on patient's general health status involving usual activity, mobility, self-care, pain, anxiety and depression, as well as visual analogue scale as a second component.	Generic
4	The World Health Organization Quality of Life Instrument WHOQOL-BREF (an abbreviated version of the WHOQOL-100). Responses illustrate experiences in the preceding 2 weeks.	Global perspective of quality of life

The Minnesota Living with Heart Failure Questionnaire (MLHFQ) includes 21 formal questions and describes physical, emotional and socioeconomic aspects of QoL (30). It is a cardiac-specific health status questionnaire, in which 13 of 21 items are summated into two subscale scores: emotional and physical. Lower scores indicate better disease-specific health status. The SF-12 is a widely used instrument consisting of 12 items that capture eight domains of self-rated health generating a physical component summary and a mental component summary scores (31, 32). The SF-12 scores are converted into a norm-based score ranging from 0 to 100, in which 50 represents the mean score of the overall US normal population, and 10 points correspond to one standard deviation (SD). Higher scores indicate better health status in the preceding month. The minimum clinically important difference for the summary scores is 2.0–2.5 points. The EuroQol (EQ-5D) is a generic instrument, commonly used in Europe and provides a five dimension scale scoring usual activity, mobility, self-care, pain/discomfort and anxiety/depression. Each dimension is scored in five levels with a lower score indicating a better QoL (28). The World Health Organization Quality of Life Instrument Abbreviated (WHOQOL-BREF, an abbreviated version of the WHOQOL-100) is designed to measure overall perspective of QoL (Table 1) (33).

In a small prospective observational study of 84 patients with VHD who underwent surgery, both the EuroQol (EQ-5D) and MLHFQ were effective for assessing QoL over a limited 6–12 week follow-up (34). A non-randomized Norwegian study of 143 patients (mean age 83 ± 2.7 years, 57% women) with AS undergoing TAVI (45%) or SAVR assessed PROMs and frailty status before and 6 month after intervention (35). The PROMs used were: (1) Medical Outcomes Study Short Form-12 questionnaire (SF-12) to assess generic aspect of self-rated health; (2) The MLHFQ to assess cardiac-specific health status; and (3) Two questions from the WHOQOL-BREF assessing the global perspective of self-reported health and QoL: “How would you rate your quality of life?” and “How satisfied are you with your health?” Patients had improved self-rated health after AVR. After TAVI, patients who were frail at baseline reported lower overall QoL and self-rated health compared with patients in the SAVR arm. The same trend was also observed at 6-months follow-up.

Frailty may affect predictions of improvement after intervention depending on its causes. If frailty is caused mainly by the VHD, patients are expected to improve after TAVI with an increase in the physical component summary score from 30.0 to 36.2 points and the mental summary component score from 42.2 to 49.6 points. After SAVR, the increase in physical component summary score was more pronounced (increased from 33.6 to 41.4), but there was no significant improvement in the mental summary component score (increased from 47.1 to 47.5 points). However, if frailty is dominated by coexistent pathology, for example chronic obstructive pulmonary disease (COPD) (36), the benefits of interventions for VHD may then be blunted. Not only will the risk of intervention be higher but also the likelihood of improvement on QoL afterwards will be lower. In situations where there is doubt about benefit, SDM exploring patients' values and preferences are even more important than usual. Some definitions include impaired cognition as a part of frailty. For patients with cognitive impairment or dementia, the concept of SDM is even more challenging and goes beyond the scope of this paper.

Nearly 20% of patients are frail at discharge following heart valve surgery and this is associated with poor self-reported health (37). International guidelines on the management of VHD recommend formal assessment of frailty status before surgery for risk stratification (38). Irrespective of the choice of valve intervention (TAVI vs. SAVR), frail patients have worse self-reported health compared with non-frail patients (39, 40). However, it is also important to highlight that following valve intervention some patients may improve in frailty status and achieve better scores on questionnaires evaluating disease-specific health status (35).

After cardiac surgery, patients with prosthetic heart valves require adequate counseling and close follow-up to make them more confident and competent to manage their own health (41). Hence, patient participation is not only essential in preoperative SDM, but also in rehabilitation programs following cardiac surgery. Experience from nurse-led clinics shows that outcomes are improved when patients are offered help to ensure guideline adherence and to identify important clinical symptoms (42, 43).

Further research focusing on values and preferences of patients with VHD, particularly AS undergoing SAVR vs. TAVI, as well as overall valve intervention vs. conservative treatment, is warranted. PROMs instruments should be used more often in research studies exploring the efficacy of intervention for patients with VHD in order to refine treatment options. In future, larger, well-designed prospective studies are needed to explore the impact of pre-intervention SDM on post-intervention outcomes including QoL, and to explore the performance of the individual PROMS instruments.

## Conclusions

Patient-centered health care places patient's autonomy, values and preferences at the core of shared decision making. Formal PROMs questionnaires encourage this process and should be used more often in daily clinical practice and in research.

## Review Criteria

- This review is based on literature and our own experience from specialist heart valve clinics.
- A comprehensive search strategy using keywords shared decision-making (SDM), patient-reported outcome measures (PROMs) and quality of life was designed.
- Bibliographic database PubMed and Embase were searched for articles published over the past 2 decades.

## Message for the clinic

- Pre-intervention SDM may improve post-intervention outcomes including quality of life.
- PROMS should be used to inform SDM for patients with heart valve disease.
- Formal PROMs questionnaires encourage communication between patient and physician and may lead to better outcomes after valve interventions.
- SDM is especially important in a clinical setting where benefit/risk is uncertain due to patients characteristics like frailty or comorbid conditions.
- In older adults, objective frailty testing is recommended to inform decision-making.
- It is important to inform patients with frailty and several comorbid conditions that repairing the valve may improve disease-specific symptoms, but may not restore the patient's functional status or quality of life.

## Author Contributions

SS, ES, AR, and JC conducted the literature search. SS, ES, and AR drafted the manuscript. JC and ØB critically revised the manuscript. All authors read and approved the final version before submission.

## Conflict of Interest

The authors declare that the research was conducted in the absence of any commercial or financial relationships that could be construed as a potential conflict of interest.

## Publisher's Note

All claims expressed in this article are solely those of the authors and do not necessarily represent those of their affiliated organizations, or those of the publisher, the editors and the reviewers. Any product that may be evaluated in this article, or claim that may be made by its manufacturer, is not guaranteed or endorsed by the publisher.
